# Miscellaneous

**Published:** 1892-04

**Authors:** 


### MISCELLANEOUS.

### LIFE AFTER FORTY.

The best half of life is in front of the man of forty, if he be any thing of a man. The work he will do will be done with the hand of a master, and not of a raw apprentice. The trained intellect does not see "men as trees walking," but sees every thing clearly and in just measure. The trained temper does not rush at work like a blind bull at a haystack, but advances with the calm and ordered pace of conscious power and deliberate determination. To no man is the world so new, and the future so fresh, as to him who has spent the early years of his manhood in striving to understand the deeper problems of science and life, and who has made some headway toward comprehending them. To him the commonest things are rare and wonderful, both in themselves and as parts of a beautiful and intelligent whole. Such a thing as staleness in life and its duties he cannot understand. Knowledge is always opening out before him in wider expanses and more commanding heights. The pleasure of growing knowledge and increasing power makes every year of his life happier and more hopeful than the last.

### TRUTH FROM THE BEYOND.

Oh, what visions of beauty and indescribable grandeur open to the astonished eyes of the liberated soul! As free and untrammeled it takes its flight through the endless realms of space, it asks: "Where have I been that I have failed to see the wonders of nature?" O beautiful death, friend and benefactor, how is thy wonderful love abused! Death is but a change, taking pain and sorrow from hearts that are weary, cooling the lips that throb and ache with burning fever. Those who are left weep because death is misunderstood. When its great mission is comprehended, fewer will be the tears and heartaches. Oh, Angel of Death! twin brother of Sleep! when thou layest thy finger on the wan lips of anguish they complain no more. Thou touchest the beating heart, and it is still.

"Beyond the clouds of sin and pain,
Where all is bright and fair,
You'll meet your much loved ones again,
Kind death will take you there."—S. B. B.

### ROYAL BAKING POWDER.

The Baking Powder that contains no injurious substance and does the business thoroughly, with no failures, is the one to rely on. Housewives are quick to discover the best, hence the more extensive sales of the "Royal." During the last year, including the contract just made under date of March 5, the Royal Baking Powder Company has supplied over 212,000 pounds, or 106 tons, of baking powder for the United States Government, and its Army and Navy officers, it being found that this is the only Baking Powder that will keep and retain its strength in the various climates to which it is sent by the Department; besides it was found to be superior to all others in leavening power by the official chemical tests, made at the instance of the Government, in the Agricultural Department, at Washington.

FUNERALS MAKE FUNERALS.—The melancholy death of the Duke of Clarence has raised the question of discontinuing the custom of standing bareheaded at a grave. It has been thought that the Duke caught a chill at the funeral of Prince Victor of Hohenlohe. The weather was bitter at the time, says the *Times*, and the wind was fierce and cutting as the Duke and his father stood bareheaded at the open grave. The strongest constitutions can hardly be expected to endure such exposure with impunity. That most of the service should be conducted in a warm chapel has been strenuously advocated. A correspondent of the London *Lancet* points out that a leaflet issued by the Council of the Funeral Reform Association, and approved by the archbishops and bishops, expressly states that mourners are not expected to do any thing imperilling their own bodily welfare. Perhaps recent events may occasion a somewhat more rational ceremonial at burials of the dead.

CURIOSITIES OF THE BIBLE.—The Bible contains 3,566,480 letters, 773,746 words. 31,173 verses, 1,189 chapters and 66 books. The word "and" occurs 46,276 times. The word "Lord" occurs 1,855 times. The word "reverend" occurs but once, which is in the 9th verse of the 11th Psalm. The middle verse is in the 8th verse of the 118th Psalm. The 21st verse of the 7th chapter of Ezra contains all the letters of the alphabet except the letter J. The longest verse is the 9th verse of the 8th chapter of Esther. The shortest verse is the 35th verse of the 11th chapter of St. John. There are no words or names of more than six syllables.

WHAT WE EAT AND DRINK.—Our analyst, says the *Confectioners' Union* (Eng.), recently went to dine at a café in the City of London, where dinners are served *à la Parisienne.* This is what he had: As an aperitive, a vermouth, rendered agreeable by the addition of sulfuric acid. A potage of tapioca made of potato starch to which copper had been added. The butter was made of calf's fat colored with lead. The roast, of inferior quality, was improved with saltpetre; and he discovered a few truffles made of pressed clay. The vinegar of the salad was seasoned with vitrol. The peas—a little too green—tasted of the copper which had given them their color. Dessert: A chocolate cream; the chocolate made of glucose, red oxide of mercury, and red ochre. For coffee he was given a mixture of horse liver roasted in the oven, black walnut sawdust, and caramel. His small glass of kirschwasser, which terminated the dinner, contained as high as twenty-two centigrammes of prussic acid to the litre. After the meal the diner had a terrible thirst; he wished to drink beer, and, accordingly, ordered some. It was a concoction of poppyheads, elder, belladonna, datura, stramonium, soda, tan bark and picric acid.

